# Structural and functional polarisation of human pancreatic beta cells in islets from organ donors with and without type 2 diabetes

**DOI:** 10.1007/s00125-020-05345-8

**Published:** 2021-01-05

**Authors:** Louise Cottle, Wan Jun Gan, Ian Gilroy, Jaswinder S. Samra, Anthony J. Gill, Thomas Loudovaris, Helen E. Thomas, Wayne J. Hawthorne, Melkam A. Kebede, Peter Thorn

**Affiliations:** 1 Charles Perkins Centre, Discipline of Physiology, School of Medical Sciences, University of Sydney, Camperdown, NSW Australia; 2 Present Address: Temasek Life-Science Laboratory, Singapore, Republic of Singapore; 3 The University of Sydney Northern Clinical School, Sydney, NSW Australia; 4 Upper Gastrointestinal Surgical Unit, Royal North Shore Hospital, St Leonards, NSW Australia; 5 Department of Anatomical Pathology, Royal North Shore Hospital, St Leonards, NSW Australia; 6 Cancer Diagnosis and Pathology Research Group, Kolling Institute of Medical Research, St Leonards, NSW Australia; 7 St Vincent’s Institute, Fitzroy, VIC Australia; 8 The University of Melbourne, Department of Medicine, St Vincent’s Hospital, Fitzroy, VIC Australia; 9 Centre for Transplant and Renal Research, Westmead Hospital, Sydney, NSW Australia; 10 Westmead Clinical School, Faculty of Health and Medicine, University of Sydney, Sydney, Australia

**Keywords:** Beta cell, Diabetes, Human, Insulin, Islet, Polarity

## Abstract

**Aims/hypothesis:**

We hypothesised that human beta cells are structurally and functional polarised with respect to the islet capillaries. We set out to test this using confocal microscopy to map the 3D spatial arrangement of key proteins and live-cell imaging to determine the distribution of insulin granule fusion around the cells.

**Methods:**

Human pancreas samples were rapidly fixed and processed using the pancreatic slice technique, which maintains islet structure and architecture. Slices were stained using immunofluorescence for polarity markers (scribble, discs large [Dlg] and partitioning defective 3 homologue [Par3]) and presynaptic markers (liprin, Rab3-interacting protein [RIM2] and piccolo) and imaged using 3D confocal microscopy. Isolated human islets were dispersed and cultured on laminin-511-coated coverslips. Live 3D two-photon microscopy was used on cultured cells to image exocytic granule fusion events upon glucose stimulation.

**Results:**

Assessment of the distribution of endocrine cells across human islets found that, despite distinct islet-to-islet complexity and variability, including multi-lobular islets, and intermixing of alpha and beta cells, there is still a striking enrichment of alpha cells at the islet mantle. Measures of cell position demonstrate that most beta cells contact islet capillaries. Subcellularly, beta cells consistently position polar determinants, such as Par3, Dlg and scribble, with a basal domain towards the capillaries and apical domain at the opposite face. The capillary interface/vascular face is enriched in presynaptic scaffold proteins, such as liprin, RIM2 and piccolo. Interestingly, enrichment of presynaptic scaffold proteins also occurs where the beta cells contact peri-islet capillaries, suggesting functional interactions. We also observed the same polarisation of synaptic scaffold proteins in islets from type 2 diabetic patients. Consistent with polarised function, isolated beta cells cultured onto laminin-coated coverslips target insulin granule fusion to the coverslip.

**Conclusions/interpretation:**

Structural and functional polarisation is a defining feature of human pancreatic beta cells and plays an important role in the control of insulin secretion.

**Graphical abstract:**

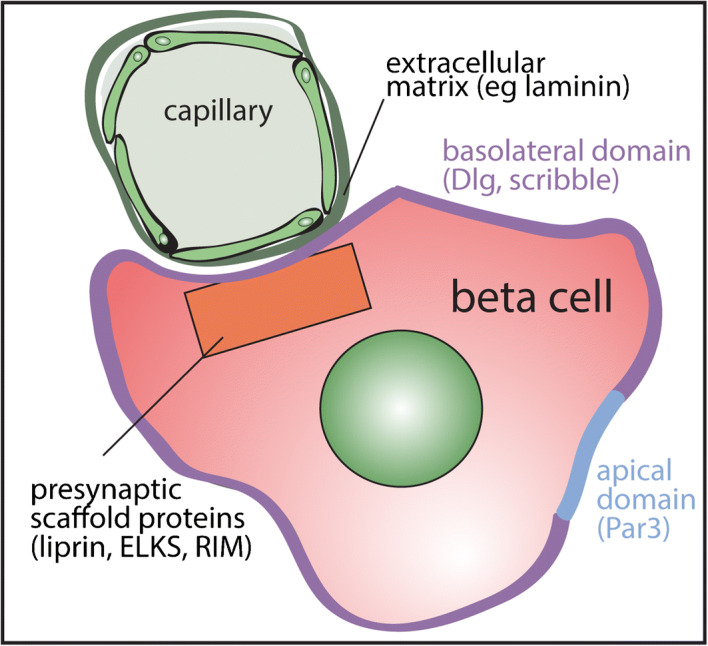

**Supplementary Information:**

The online version contains peer-reviewed but unedited supplementary material available at 10.1007/s00125-020-05345-8.

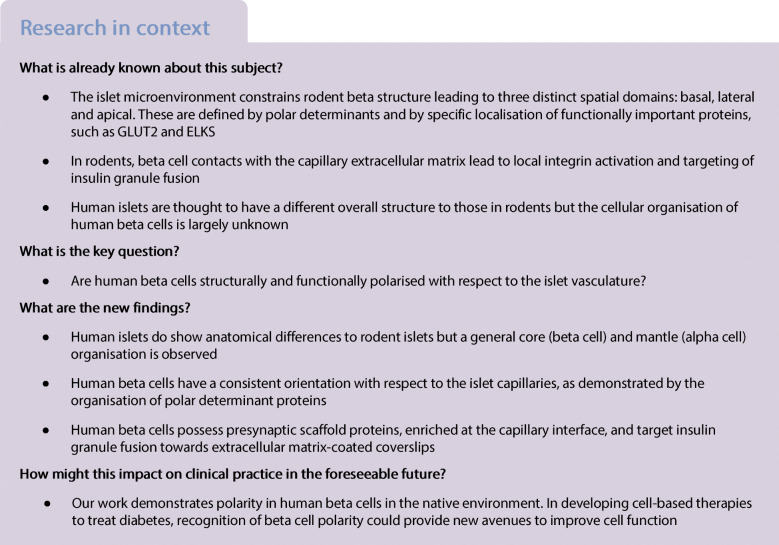



## Introduction

Understanding the functions of human pancreatic islets underpins future approaches to treating diabetes [1]. One knowledge gap is determining beta cell structure and function within native human islets [2]. There is controversy around the overall architecture of human islets [3, 4] and little is known about the subcellular organisation of endocrine cells.

In terms of islet architecture, some studies indicate that human islets have an alpha cell mantle and beta cell core, similar to rodent islets [5], with a multi-lobular morphology in larger islets [3, 6]. However, other reports suggest either a random arrangement of different endocrine cells [7] or a laminar, folded structure [4]. Another important aspect of cell organisation is the endocrine cell contacts with the islet capillary bed [8, 9]. In rodents, essentially every beta cell contacts a capillary [10] and the capillary extracellular matrix exerts significant effects on beta cell function [11–13]. In contrast, in human islets, one study suggests a subpopulation of beta cells may not touch the capillaries [14], raising the possibility that some beta cells behave differently, consistent with current ideas that beta cell heterogeneity is important to islet function [15, 16].

In terms of subcellular organisation, older observations suggested beta cell polarity in rodents [17, 18] with the impact of polarisation increasingly being recognised [10, 19]. Rodent beta cells express proteins that maintain cell polarity, such as liver kinase B1 (LKB1) [20] and polarity determinant proteins, including discs large (Dlg) and partitioning defective 3 homologue (Par3), in polar regions analogous to epithelial cells [10]. This polarity is consistently organised with respect to blood vessels with the basal region facing the capillaries and the apical region opposite [10]. These domains are functionally important; for example the basal membrane domain facilitates the targeting of insulin exocytosis to the blood vessels [10, 21].

The machinery of insulin granule fusion is known [22] but the structural mechanisms that organise and control insulin granule delivery to the cell surface are currently unclear. Multiple presynaptic complex proteins, including Rab3-interacting protein (RIM2), piccolo and liprin, are expressed in mouse islets [23]. Knockdown of these proteins, including RIM2 and ELKS, impairs insulin secretion [24, 25]. These proteins are enriched at the beta cell–vasculature interface in mice [21] and functional studies demonstrate preferential insulin exocytosis at the vascular face [21], suggesting that a presynaptic-like complex or beta cell ‘synapse’ controls insulin granule delivery to the cell surface [21] in mechanisms similar to neuronal synapses. All these studies focus on rodents and there is little knowledge of the spatial organisation of the structure of human beta cells or evidence for functional polarisation.

On the basis of the work on rodent islets we hypothesised that human beta cells would also show structural and functional polarity. However, as outlined above, some studies on human islet structure indicate that human beta cells have a different relationship to the islet capillaries. Since, in mice, these contacts with the capillary extracellular matrix drive beta cell organisation, it is important to determine the arrangement of beta cells within human islets and to identify their subcellular structure. To best preserve native islet structure we used pancreatic slices [26], which were rapidly fixed and assessed with high resolution 3D immunofluorescence. To understand the overall structure of the human islets we mapped the arrangement of alpha and beta cells and the islet capillaries. Then, to understand the subcellular organisation of beta cells, we identified their contact points with the islet capillaries and determined the distribution of key proteins across the cells. Functional experiments were not possible in slices, therefore we used isolated beta cells. We cultured the cells on extracellular matrix proteins, stimulated with glucose and assessed the spatial arrangement of insulin granule fusion.

## Methods

### Human pancreatic tissue sources

Tissue was sourced from pancreatic tumour resections or cadaveric donors. Tumour resections were performed at the Royal North Shore Hospital (St Leonards, NSW, Australia) and tissue was collected with patient consent, approved by the Northern Sydney Local Heath District Human Research Ethics Committee. Fixed pancreatic sections were from the JDRF Network for Pancreatic Organ donors with Diabetes (nPOD) tissue bank, approved by the Human Research Ethics Committee at the University of Sydney.

Cadaveric donor tissue and islets were sourced from St Vincent’s Institute (Fitzroy, VIC, Australia). Informed consent was acquired, and the study approved by the Human Research Ethics Committee at the University of Sydney. Cadaveric donor islets were also sourced from Westmead Hospital (Sydney, NSW, Australia). Informed consent was acquired, and this study was approved by the Western Sydney Local Heath District Human Research Ethics Committee.

Information on pancreatic tissue donors and islet donors are listed in electronic supplementary material (ESM) Tables 1 and 2. Detailed information on the human islet preparations is listed in the Human islet checklist in the ESM. Background information on diabetic tissue donors is listed in ESM Table 3.

### Pancreatic tissue fixation and sectioning

Tissue was fixed using 4% paraformaldehyde at 4°C for 2.5 h. Tissue was washed before storage at 4°C in PBS supplemented with 0.01% sodium azide. Sectioning of fixed tissue was performed as described by Marciniak et al [26]. Fixed tissue was mounted in 1.5% low melting point agarose and cut into 150 μm sections using a vibratome.

### Pancreatic slice staining

Sections were stained as described by Meneghel-Rozzo et al [27] and incubated in blocking buffer (3% BSA, 3% donkey serum, 0.3% Triton X-100) for 4 h at room temperature, and then in primary antibody at 4°C overnight. Sections were washed in PBS and secondary antibody and DAPI incubations were for 5 h at room temperature. After washing, sections were mounted using ProLong Diamond Antifade (Thermo Fisher Scientific) and imaged on a Leica SP8 confocal, 63× objective (Leica Microsystems, Germany). Antibody details are listed in ESM Table 4.

### Protein coating of coverslips

Glass coverslips were coated using 5 μg/μl laminin-511 (Biolamina, Sweden) and incubated at 4°C for 16 h.

### Human islet culture, dispersion, fixation and staining

Islets were cultured in RPMI-1640 supplemented with 10% FBS and 100 U/ml penicillin plus 0.1 mg/ml streptomycin at 37°C and 5% CO_2_. Islets were dispersed (TrypLE express, Thermofisher Scientific, Australia) and cultured on laminin-511-coated coverslips for 20 h before fixation with 4% paraformaldehyde at room temperature for 15 min.

Coverslips were incubated in blocking buffer (3% BSA, 3% donkey serum, 0.1% Triton X-100) for 45 min and then with primary antibody for 2 h at room temperature. Coverslips were washed in PBS. Secondary antibody and DAPI incubations were 45 min at room temperature. After washing, coverslips were mounted and imaged using a Leica SP8 confocal microscope.

### Live-cell imaging

Dispersed cells were cultured overnight on laminin-511 in RPMI-1640 media. Prior to imaging, cells were incubated in Na-rich extracellular solution (140 mmol/l NaCl, 5 mmol/l KCl, 1 mmol/l MgCl_2_, 2.5 mmol/l CaCl_2_, 5 mmol/l NaHCO_3_, 5 mmol/l HEPES, pH 7.4) containing 3 mmol/l glucose for 30 min. Two-photon imaging was performed at 37°C with exocytic events recorded as the entry of extracellular sulforhodamine B dye (8 mmol/l) (Merck, Australia) into fused granules upon 15 mmol/l glucose stimulation. For focal adhesion kinase (FAK) inhibition cells were treated with 1 μmol/l Y15 (Merck, Australia) for 30 min prior to imaging. Imaging was performed using a custom-built multiphoton microscope based around an Olympus IX70 and controlled by ScanImage 3.8 software.

### Quantification and statistical analysis

**Fig. 1:**
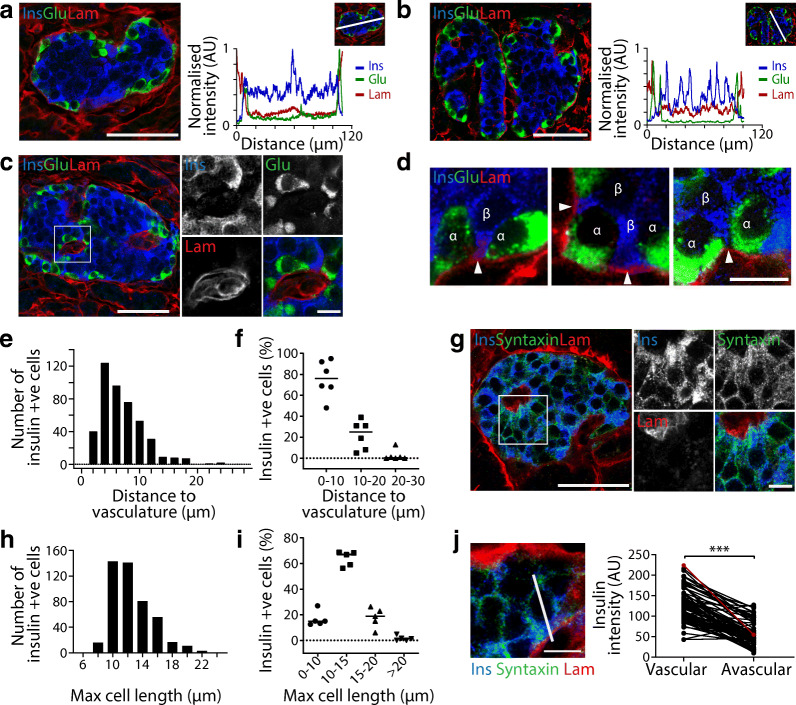
Analysis of beta cell arrangement with respect to the vasculature. Human islets were fixed and the predominant cell types and vasculature were examined. (**a**–**d**) Representative images demonstrating that human islets are arranged with an alpha cell (glucagon, green) mantle and beta cell (insulin, blue) core and surrounded by a laminin capsule (red). Both (**a**) single and (**b**) multi-lobular islets retain the same structure within lobes. Linescans across a whole islet lobe show a core–mantle arrangement with bright glucagon signal close to laminin and insulin signal situated in the middle. (**c**) Human islets stained with insulin (blue) and glucagon (green) display a heterotypic cell arrangement along the vasculature (laminin, red). (**d**) Zoomed-in images showing beta cells (β) stretching through alpha cells (α) to contact the vasculature. White arrows indicate beta cell contact with vasculature. Data in (**a**–**d**) are representative of 14 islets from 3 donors. (**e**, **f**) Analysis of the distance from beta cell to the vasculature indicating that most cells are within 10 μm of the vasculature. (**e**) A frequency histogram showing data from individual cells (*n*=448 cells); (**f**) data presented using islet averages. Data in (**e**, **f**) is representative of 3 donors; *n*=6 with 2 islets analysed per donor. (**g**) Representative images demonstrating that beta cells (blue) elongate to make contact with islet blood vessels (red); beta cell membranes were visualised using syntaxin (green). (**h**) A histogram showing maximum beta cell length from individual cells (*n*=468 cells). (**i**) Maximum beta cell length presented using islet averages. Data in (**h**–**i**) are representative of 3 donors; 1–2 islets analysed per donor. (**j**) Insulin immunostaining has a higher intensity towards the vasculature as demonstrated by linescan analysis at the vascular and avascular face of beta cells. The red dataset on the graph represents the beta cell from the image. Data are representative of 3 donors; *n*=83 cells analysed using 1–2 islets per donor. Data represent mean ± SEM in (**f**, **i**). Scale bars: 50 μm on whole islet images; 10 μm on zoomed-in images. Data were collected from both pancreatomised and cadaveric donor samples. ***p<0.001. Glu, glucagon; Ins, insulin; Lam, laminin; +ve, positive

Image analysis was performed using Fiji (ImageJ) [28] and Metamorph 7.8 (Molecular Devices, USA). Graphs were produced using GraphPad Prism v7.02 (GraphPad Software, USA). Linescan analysis measured the fluorescence intensity along a line on a confocal image and was performed using Metamorph. In Fig. 1, a 150 pixel thick line was drawn across the islet lobe, fluorescent intensities were analysed along the line. Intensities were normalised to the brightest area and graphed. To determine beta cell distance from blood vessels, the beta cell (insulin) nucleus midpoint was determined using DAPI signal in *z*-stacked images. A straight line was drawn from the nucleus midpoint to the nearest capillary (laminin and CD31/von Willebrand factor [VWF]). To determine beta cell maximal length, beta cell membranes were visualised using syntaxin 1A. The plane displaying the maximal length was determined and a straight line drawn from one edge of the cell to the opposite edge, through the nucleus. For insulin intensity, beta cells adjacent to the vasculature were identified and a line was drawn from the vascular to avascular face. Using syntaxin 1A to define cell boundaries, the average insulin intensity was measured across the line and extending into the cell for 2 μm from each face.

**Fig. 2:**
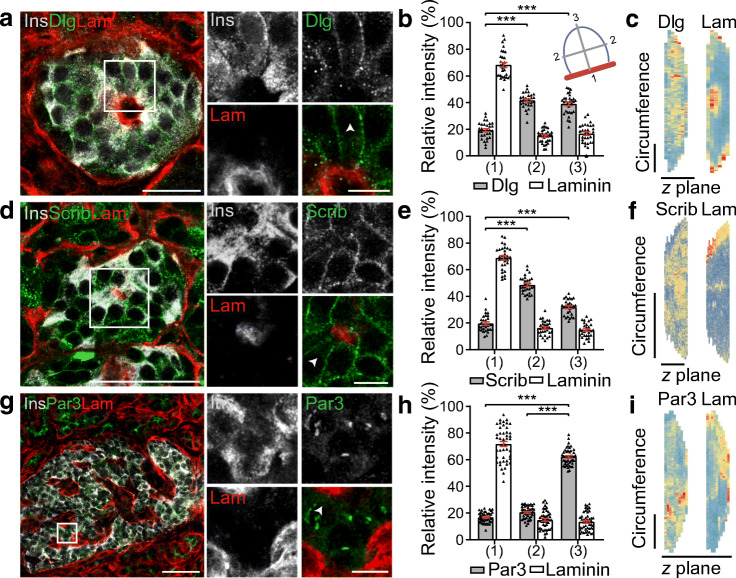
Human beta cells are organised structurally using conventional polarity determinant proteins. (**a**) Immunofluorescence images of an islet within a pancreatic slice demonstrating that in beta cells (insulin, grey), Dlg is enriched (green) around the membrane including the vascular face (labelled with laminin, red) and lateral domains. (**b**) Fluorescence intensity was assessed at plasma membrane domains shown in the cell diagram 1: vascular face; 2: lateral; 3: avascular face. Data are representative of 2 donors; *n*=30 cells with 15 cells from 3 islets per donor. (**c**) 3D heat map representation (low, blue; high, red) of fluorescence intensity using cell circumference fluorescence at each *z*-stack, demonstrates that Dlg is dispersed across the cell membrane. (**d**) Representative immunofluorescence images (insulin, grey; scribble, green; laminin, red) and (**e**) fluorescence intensity analysis show scribble is similarly distributed across the cell membrane. Data are representative of 2 donors; *n*=30 cells with 15 cells from 3 islets per donor. (**f**) 3D heat map of fluorescence intensity shows an enrichment of scribble on the cell membrane. (**g**) Representative immunofluorescence images (insulin, grey; Par3, green; laminin, red) and (**h**) fluorescence intensity analysis show Par3 is enriched in a discrete avascular domain away from the capillaries. Data are representative of 3 donors; *n*=45 cells with 15 cells from 3 islets per donor. (**i**) 3D heat map of fluorescence intensity demonstrates that Par3 signal is enriched on the cell membrane away from the vascular face. The arrowheads in (**a**, **d**, **g**) indicate the cell used in the heat maps in (**c**, **f**, **i**). Scale bars: 50 μm on whole islet images; 10 μm on zoomed-in images and 3D heat maps. Data represent mean ± SEM in (**b**, **e**, **h**). Data were collected from both pancreatomised and cadaveric donor samples. ***p<0.001. Ins, insulin; Lam, laminin; Scrib, scribble

**Fig. 3:**
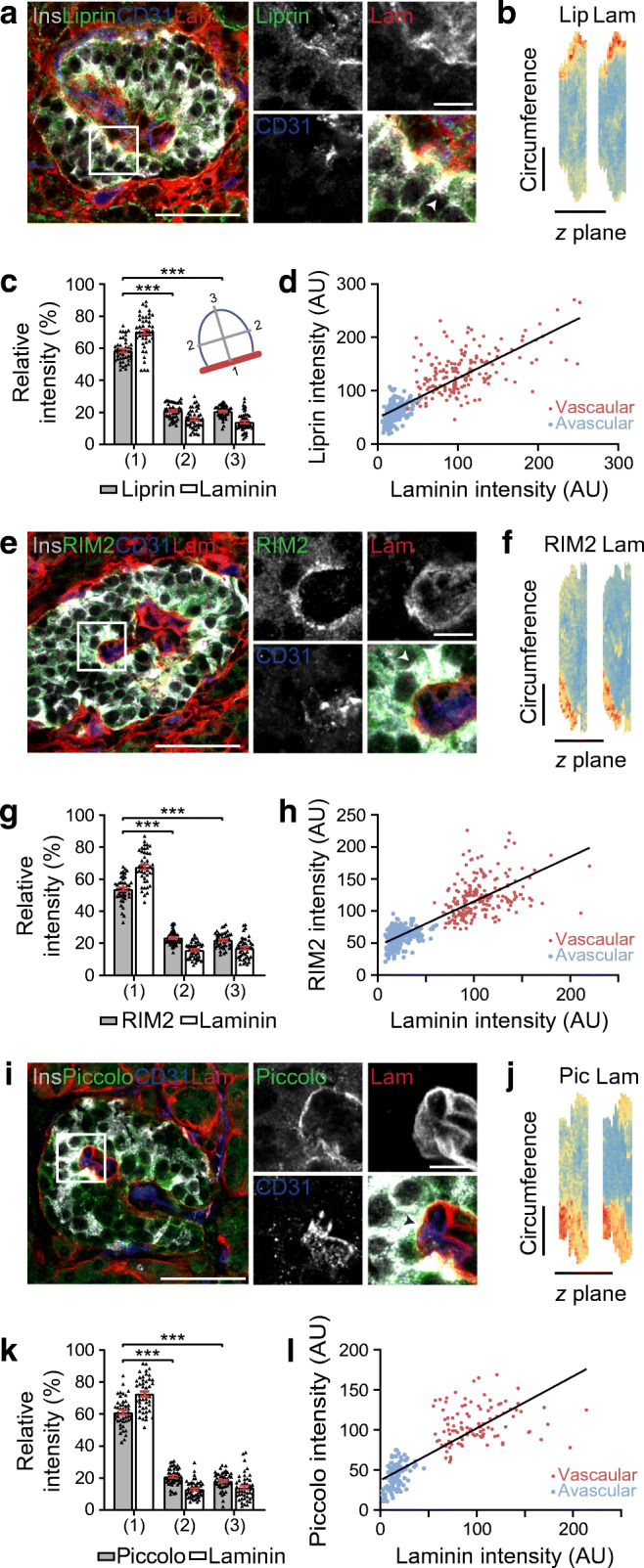
Synaptic proteins liprin, RIM2 and piccolo are enriched along the vascular face of beta cells making contact with intra-islet blood vessels. (**a**) Representative images of a pancreatic islet demonstrating beta cells (insulin, grey) have an enrichment of the presynaptic protein liprin (green) along the contact face with intra-islet blood vessels (laminin, red; CD31, blue). (**b**) 3D heat map representation of fluorescence intensity (low, blue; high, red) using cell circumference fluorescence analysis at each *z*-plane in the stack demonstrates enrichment of liprin along the vascular face as shown by points of brightest liprin and laminin signal (red) being co-located. (**c**) Fluorescence intensity assessed at plasma membrane domains shown in the cell diagram 1: vascular face; 2: lateral; 3: avascular face, shows significant differences across the domains, as indicated. Data from 3 donors; *n*=45 cells, 15 cells from 3 islets per donor. (**d**) Regional analysis of fluorescence intensity of liprin staining at the vascular and avascular faces of beta cells shows a consistent relationship where higher laminin signals, indicative of the vascular face, are accompanied by higher liprin signals. Black line represents the line of best fit, *r*^2^= 0.63; *n*=168 cells, 3 donors, 3 islets per donor. (**e**) RIM2 is also enriched along the vascular face of beta cells. Representative images (insulin, grey; RIM2, green; CD31, blue; laminin, red). (**f**) 3D heat map representing fluorescence intensity demonstrates enrichment of RIM2 along the vascular face identified with laminin staining. (**g**) Analysis of fluorescence intensity shows significant differences across the domains, as indicated. Data from 3 donors, *n*=45 cells with 15 cells from 3 islets per donor. (**h**) Regional analysis of RIM2 staining shows a consistent relationship with laminin staining. *r*^2^= 0.65; *n*=198 cells, 3 donors, 3 islets per donor. (**i**) Piccolo is also enriched along the vascular face of beta cells. Representative immunofluorescence images (insulin, grey; piccolo, green; CD31, blue; laminin, red). (**j**) 3D heat map representing fluorescence intensity demonstrates an enrichment of piccolo along the vascular face. (**k**) Analysis of fluorescence intensity shows significant differences across the domains, as indicated. Data from 3 donors; *n*=45 cells, 15 cells from 3 islets per donor. (**l**) Regional analysis of piccolo staining shows a consistent relationship with laminin staining. *r*^2^=0.65; *n*=102 cells, 3 donors, 3 islets per donor. The arrowheads in (**a**, **e**, **i**) show the individual cells used in the heat map. Scale bars: 50 μm on whole islet images; 10 μm on zoomed-in images and 3D heat maps. Data represent mean ± SEM in (**c**, **g**, **k**). Data were collected from both pancreatomised and cadaveric donor samples. *** *p*<0.001. Ins, insulin; Lam, laminin; Lip, liprin; Pic, piccolo

**Fig. 4:**
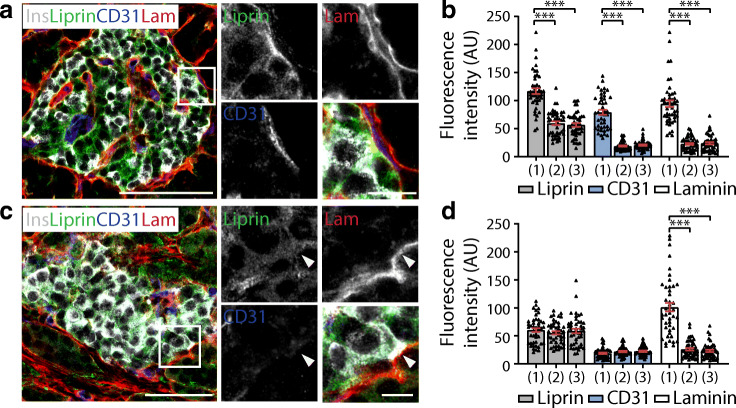
Presynaptic protein liprin is enriched along the vascular face of beta cells making contact with peri-islet blood vessels. (**a**) Representative images of a pancreatic islet demonstrating that beta cells (insulin, grey) have enrichment of synaptic protein liprin (green) along the contact point with blood vessels surrounding the islet (laminin, red; CD31, blue). (**b**) Liprin fluorescence intensity at plasma membrane domains (1: vascular face; 2: lateral; 3: avascular face) shows enrichment of liprin at the vascular face identified with CD31 enrichment. Data are representative of 3 donors; *n*=45 cells with 15 cells from 3 islets per donor. (**c**) Liprin is not enriched along the islet capsule of beta cells on the outer edge of islets. White arrows show the beta cell–laminin contact point with the capsule. (**d**) Analysis of fluorescence intensity shows no regional enrichment of CD31 and no localisation of liprin. Data are representative of 3 donors; *n*=45 cells with 15 cells from 3 islets per donor. Scale bars: 50 μm on whole islet images; 10 μm on zoomed-in images. Data represent mean ± SEM in (**b**, **d**). Data were collected from both pancreatomised and cadaveric donor samples. ***p<0.001. Ins, insulin; Lam, laminin

For fluorescence intensity graphs (Figs 2, 3, 4, 6) beta cells adjacent to capillaries were selected randomly. The intensity at three different plasma membrane domains (1: vasculature contact; 2: lateral; and 3: avascular) was averaged along the line and graphed.

3D heat maps of individual beta cells (Figs 2, 3, 6) show the surface distribution of proteins of interest. For each *z*-plane the cell outline (circumference) was drawn and fluorescence intensity along the line determined. These intensities were colour-coded in Microsoft Excel (Microsoft, USA) to indicate regions of high (red) or low (blue) fluorescence intensity. This was repeated for each subsequent *z*-plane to build up a 3D map of fluorescence intensity across the whole cell surface (see Fig. 2c).

**Fig. 5:**
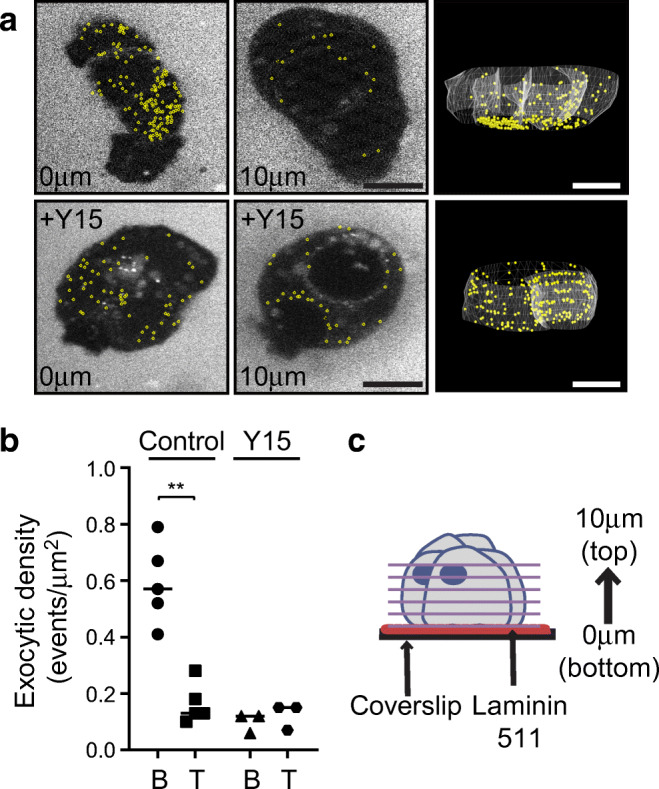
3D live cell two-photon imaging demonstrates glucose-dependent granule fusion is targeted towards ECM-coated coverslips. Islet cell clusters cultured on laminin-511-coated coverslips were stimulated with 15 mmol/l glucose and granule fusion events were recorded. (**a**) Representative images of exocytosis events (yellow) at the interface with the coverslip (0 μm) and at 10 μm above the coverslip. 3D models of all fusion events are also included. (**b**) Quantification of fusion events. B (bottom) represents the plane cells contact the coverslip (0 μm) and T (top) is the area of the cells above the coverslip (2–10 μm); **p=0.001. For control *n*=5 dispersed islet cell clusters obtained from 2 islet preparations, for Y15 treatment *n*=3 from 3 islet preparations. (**c**) Diagrammatic representation of the imaging method. Scale bars, 10 μm. Data represent mean ± SEM

**Fig. 6:**
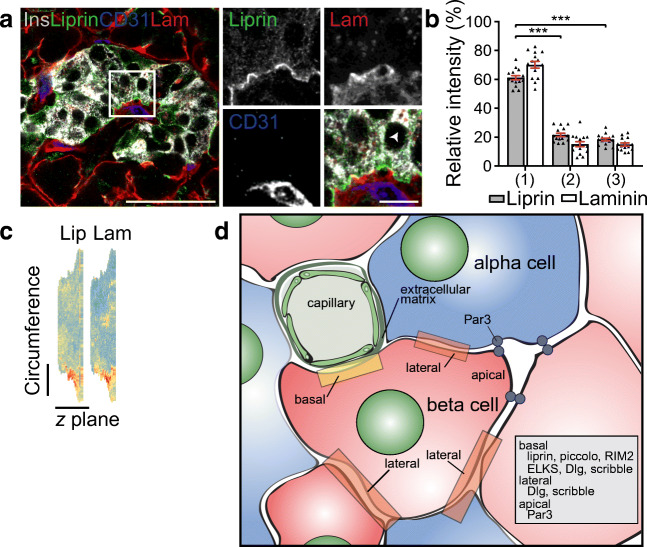
Type 2 diabetic islets maintain enrichment of presynaptic proteins at the vasculature. (**a**) Representative images demonstrating that beta cells (insulin, grey) have an enrichment of synaptic protein liprin (green) at the interface with blood vessels (laminin, red; CD31, blue); representative of 3 donors. (**b**) Fluorescence intensities of liprin at plasma membrane domains. Data analysed from 1 donor (Sample 6); *n*=15 cells from 3 islets. The nPod samples were partly degraded and not suitable for quantitative analysis. (**c**) 3D heat map of fluorescence intensity demonstrates an enrichment of liprin along the vascular face represented by the points of brightest liprin and laminin signal (red) being co-located. The cell used is shown by an arrowhead in (**a**). (**d**) Cartoon summary of polar organisation of human beta cells, which are organised with respect to the fenestrated capillaries. Scale bars: 50 μm on whole islet images; 10 μm on zoomed-in images and 3D heat maps. Graph presented as mean ± SEM. ***p<0.001 Ins, insulin; Lam, laminin; Lip, liprin

Statistical analyses used GraphPad Prism. A paired two-tailed student’s *t* test was used to analyse insulin intensity in Fig. 1j, regional analysis of fluorescence intensity in Fig. 3d,h and l and the two-photon data in Fig. 5. In Figs 2, 3, 4, 6 and ESM Fig. 5 fluorescence intensity was analysed using one-way ANOVA followed by Sidak’s multiple comparison test. Samples were not randomised or blinded, owing to the nature of the tissue collection, with each sample being collected and processed individually.

## Results

We paraformaldehyde-fixed samples of human pancreas, from patients undergoing partial pancreatectomy or cadaveric donors, sliced the tissue [26] into 150 μm sections, immunostained for proteins of interest and imaged with 3D confocal microscopy. This approach provides excellent preservation of tissue structure that was consistent for all sources of material.

### Most beta cells contact the islet capillaries

We first characterised the distribution of endocrine cells and their relationship to islet capillaries, both contentious issues in the field. We co-stained basement membrane proteins, laminin and nidogen-1, showing that they stain the same structures (Fig. 1, ESM Fig. 1). In single-lobed islets we observed an alpha cell mantle and beta cell core structure (Fig. 1a), which is maintained in each lobe of multi-lobular islets (Fig. 1b). Alongside laminin-labelled capillaries we observed alpha and beta cells, with beta cells reaching through alpha cells to contact capillaries (Fig. 1c,d). It has been suggested that human islets contain fewer capillaries than in rodents [29], which might impact the proportion of beta cells that contact capillaries. To determine this, we measured the distance from the centre of each beta cell nucleus to the nearest capillary (laminin and CD31) using similar methods to Cohrs et al [14]. Similarly to these authors’ findings, most beta cells were <10 μm from a capillary (Fig. 1e,f). This distance is within the diameter of most cells. However, interestingly, further analysis of cell outline, using syntaxin 1A as a surface marker, indicated that human beta cells were elongated, rather than spherical (Fig. 1g–i), as has been shown before [4]. Analysis of maximal beta cell length showed that >61% of cells were between 10 and 15 μm (Fig. 1h,i). We acknowledge that a small subpopulation of beta cells might be remote from capillaries; however our analysis indicates that most human beta cells do contact capillaries.

We then investigated whether vasculature contact influenced insulin distribution within each beta cell, an idea suggested by a recent study [19]. Insulin distribution, determined by measuring fluorescence intensity along a line drawn perpendicular to the vascular face shows a significantly higher intensity at the vascular face of beta cells (Fig. 1j*p* < 0.001, *n* = 83 cells). Together our analysis indicates that most beta cells contact a capillary and show polarised insulin distribution with enrichment at the vascular face.

### Beta cells maintain consistent polarity with respect to the vasculature

We next tested whether human beta cells possess the structural polarity components that underlie distinct plasma membrane domains found in mice [10]. We immunostained for the polarity determinants Dlg, scribble and Par3, and the capillary basement membrane with laminin. Basolateral polarity determinant proteins Dlg and scribble are enriched around the surface membrane of beta cells and at the interface with the capillary (Fig. 2a–f). 3D heat maps, created by measuring the fluorescence intensity of the cell circumference over sequential *z*-planes, further support the enrichment of Dlg and scribble in the basolateral domains (Fig. 2c,f). In these images, Dlg and scribble are particularly enriched on the lateral surfaces (Fig. 2a–f, comparing vascular with lateral fluorescence intensity, Dlg was enriched *p* < 0.001, *n* = 30 cells and scribble was enriched *p* < 0.001, *n* = 30 cells). Localisation of the apical polarity determinant, Par3, shows significant enrichment in regions away from the vasculature (Fig. 2g–i, comparing vascular with avascular fluorescence intensity, Par3 was enriched *p* < 0.001, *n* = 45 cells). Immunostaining of these polarity determinants in isolated human beta cells shows that these proteins are specifically present in beta cells (ESM Fig. 2). These results demonstrate that human beta cells are polarised and this polarisation was consistent for all beta cells in the islet.

### Evidence for the presence of a beta cell ‘synapse’ in human beta cells

In mouse islets there is an enrichment of presynaptic scaffold proteins at the beta cell–vasculature interface [21], which is linked with the spatial targeting of insulin granule fusion [11]. We therefore tested whether human beta cells possess similar structures.

Immunofluorescence staining revealed strong enrichment of liprin (Fig. 3a), RIM2 (Fig. 3e) and piccolo (Fig. 3i) in the beta cells at the vasculature interface (labelled with CD31 and laminin). The 3D mapping (Fig. 3b,f,j) and analyses of fluorescent intensities in regions across the cell (Fig. 3c,g,k) were consistent with enrichment of all three presynaptic scaffold proteins at the vascular face (*p* < 0.001 liprin *n* = 45 cells; RIM2 *n* = 45 cells; piccolo *n* = 45 cells;). Image intensity analysis comparing liprin, RIM2 and piccolo distribution at the vascular with the avascular face (Fig. 3d,h,l) showed each were significantly enriched in the vascular region (liprin, *p* < 0.001, *n* = 168 cells; RIM2, *p* < 0.001, *n* = 198 cells; piccolo, *p* < 0.001, *n* = 102 cells). Immunostaining of isolated human beta cells shows that these proteins are specifically expressed by beta cells (ESM Fig. 3). Owing to antibody incompatibility we were unable to co-stain all presynaptic markers but did confirm that liprin and ELKS are co-enriched at the vascular interface (ESM Fig. 4). These results indicate that a presynaptic-like complex exists in human beta cells and is localised at the vascular interface.

### Beta cells orientate presynaptic machinery in response to peri-islet vasculature

In addition to intra-islet capillaries, peri-islet blood vessels, are observed (CD31, Fig. 4). Interestingly, we observed that beta cells contacting these peri-islet capillaries also orientate themselves with respect to these blood vessels. Beta cells contacting the peri-islet capillaries (CD31 and laminin) have polarised enrichment of synaptic scaffold proteins at the capillary interface, including liprin (Fig. 4a,b; *p* < 0.001, *n* = 45 cells), RIM2 and piccolo (ESM Fig. 5a, b and e, f). In contrast, beta cells contacting just the islet capsule, as shown by laminin-only staining, show no regional enrichment of the presynaptic proteins liprin (Fig. 4c,d), RIM2 or piccolo (ESM Fig. 5c, d and g, h). Note the laminin in the capsule and capillaries is identified using a laminin β1-specific antibody. This work demonstrates that beta cells recognise a capillary or endothelial cell-specific factor to establish local presynaptic protein complexes.

### Culture of beta cells on basement membrane proteins induces targeted insulin granule fusion

The data above indicate that human beta cells polarise and orientate presynaptic scaffold proteins to the capillary interface. Whether this targets insulin granule fusion to the capillaries is not known and, to date, we have not been able to image live human slices. However, in mice we have recapitulated cell orientation, in an integrin-dependent manner, by culturing isolated cells onto laminin (a major islet matrix protein) coated coverslips [11]. Therefore, to determine whether human beta cells can orientate and target insulin secretion we dispersed human islets and cultured the cells on laminin-coated coverslips. 3D live-cell two-photon microscopy identified granule fusion events [30, 31]. Cells were bathed in an extracellular fluorescent dye (sulforhodamine B) and granule fusion events were identified by the entry of dye into each fusing granule [32]. Each fusion event is characterised by a sudden, spatially discrete rise in fluorescence followed by a slower decay as the granule collapses into the membrane [11]. Cells were stimulated with 15 mmol/l glucose and imaged in 3D over time with granule fusion events identified in space and time. Human beta cells cultured on laminin-coated coverslips showed a significant bias of granule fusion events towards the coverslip (Fig. 5, *n* = 5, *p* = 0.001).

Mouse beta cells target granule fusion in a focal adhesion/integrin-dependent manner that can be blocked by inhibition of focal adhesion kinase [11], a key downstream component of integrin activation [33]. To assess whether this mechanism is present in human beta cells, cells were treated with the FAK inhibitor Y15, which abolished the targeting of exocytosis events to the laminin-coated coverslip (Fig. 5b, *n* = 3). We conclude that focal adhesion activation drives human beta cell orientation and targets insulin secretion.

### Type 2 diabetic islets show vascular enrichment of presynaptic machinery

Our work reveals polarity in human beta cells and spatial enrichment of presynaptic proteins at the capillary interface—both of which are likely to be important for cell function. Given that beta cell failure is a key feature of type 2 diabetes [34] we sought to determine whether this cellular organisation is disrupted in disease. We obtained tissue from patients identified with type 2 diabetes from Australia (Sample 6) and material from the nPOD tissue bank. Immunofluorescence staining from either source of type 2 diabetic patient material demonstrated enrichment of presynaptic protein liprin at the beta cell–vasculature interface (Fig. 6). This enrichment was identified using fluorescence intensity distribution analysis and 3D heat maps. The preservation of this relationship even in disease suggests this subcellular arrangement is fundamental to beta cell structure. However, this qualitative imaging data does not rule out possible alterations in protein expression or the possibility that other proteins, associated with granule fusion, may be affected in disease.

## Discussion

Our analysis of the 3D arrangement of endocrine cells in human islets shows some intermixing of cell types and a general core–mantle structure with alpha cells on the outside of single-lobed islets or individual lobes of multi-lobed islets. Most beta cells contact the intra-islet or peri-islet capillaries. At the subcellular level, our work reveals that individual beta cells in human islets are polarised, as shown by classical polarity determinants, with a basal pole at the capillary interface and apical pole opposite. The beta cell capillary interface is enriched in presynaptic scaffold proteins suggesting insulin granule exocytosis is targeted to this area. Consistent with this, isolated cells cultured on laminin-coated coverslips target insulin granule fusion to the coverslip in a focal adhesion-dependent manner. These findings prove structural and functional polarity of human beta cells (see Fig. 6d).

### Distribution of endocrine cells in human islets

The intermixing of alpha and beta cells in human islets is distinct from rodent islets [35]. In rodents most beta cells are in the islet core and contact other beta cells [10, 35]. In contrast, in humans there is a greater proportion of alpha cells compared with beta cells and the beta cells commonly contact alpha cells [4, 7]. The core–mantle structure of rodent islets has been proposed to be significant for paracrine communication from beta cells to alpha cells [36]. However, the blood flow (core to mantle) required for this is contested [37] and, now in the light of the mixing of cell types in human islets, it seems unlikely that the relative location of different endocrine cells plays a specific role in islet paracrine signalling [8].

### Human beta cell–blood vessel contacts

In mouse islets almost all beta cells contact the capillaries [10, 18] and target insulin granule fusion to this vascular face [21]. In contrast, reports that the proportion of beta cells contacting the vasculature in human islets is lower than in mouse islets [14] might indicate that sub-populations of human beta cells do not secrete. However, this conclusion was made by measuring the distance from the nucleus midpoint to the nearest capillary, suggesting that any beta cells with a distance of more than 10 μm from the capillary (just under 40% of beta cells) were unlikely to make vascular contact [14]. We repeated the same analysis and obtained the same result. However, we determined that beta cells are elongated and most cells (>61%) have a long axis of between 10 and 15 μm (Fig. 1). Therefore, we suggest that most human beta cells do contact the vasculature, potentially by stretching around alpha cells, as we and others show [4].

Our observation that beta cells are orientated with respect to peri-islet capillaries suggests a functional interaction where cells target secretion to these blood vessels. It is unclear whether this is distinct from that of intra-islet secretion or whether it serves another function, such as a local paracrine effect. It is also unclear what specifically the beta cells are sensing from the capillaries that is provoking the orientation.

### Human beta cells polarise with respect to the vasculature

In mouse islets beta cells align along the capillaries and form a basal surface as indicated by the enrichment of Dlg and scribble, a lateral surface, also enriched in these markers and an apical region identified using Par3 and the primary cilia [10]. It is unknown if these polarity determinants functionally define these regions, as they do in epithelial cells. However, we do know that in mouse islets the glucose transporter, GLUT2 is located at the lateral domain and that tight junctions [11, 21, 38], identified with zonula occludens 1 (ZO-1) are located at the apical domain, suggesting that polarisation does have functional significance for the cell. The data we now present for human beta cells is consistent with this model and shows an identical location of Dlg, scribble and Par3.

### Polarisation of presynaptic scaffolds and targeting of insulin secretion

An example of polar organisation in mouse islets is the enrichment of presynaptic scaffold proteins at the vascular interface of beta cells [21]. Here, we show an identical organisation of presynaptic scaffold proteins where the beta cells contact the capillary in human islets. In the mouse, one mechanism driving this organisation is integrin dependent and, in vitro, inhibition of downstream pathways such as FAK disrupts targeting of insulin granule fusion to the sites where beta cells contact extracellular matrix proteins [11]. We now show the same phenomena in human beta cells. While further experiments are needed, in particular live-cell 3D imaging in human pancreatic slices, our work shows that human beta cells could target insulin granule fusion to the capillary face in a mechanism dependent on integrins and the activation of focal adhesions [33]. This model would thus require that insulin exits the beta cells, crosses the basement membrane [39] and then enters the capillary.

### Role of polarisation in disease

Islets from donors with type 2 diabetes showed the same enrichment of presynaptic scaffold proteins, at the vascular face, as islets from non-diabetic individuals. The preservation of this structural arrangement in islets from diabetic donors suggests this arrangement and the associated mechanisms of secretion are fundamentally important. This indicates that the cells might maintain secretory competence and may explain the restoration of insulin secretion by sulfonylureas and glucagon-like peptide 1 (GLP-1) based treatments in type 2 diabetic patients [40]. We note here that we have not performed functional studies from type 2 diabetic samples and, although the synaptic scaffold proteins seem to be correctly positioned, we cannot rule out functional mistargeting of insulin secretion. Consistent with this, glucotoxicity does block targeting of insulin granules in the mouse [11].

### Conclusion

Our data demonstrate that, despite differences in human and rodent islet architecture, most human beta cells do contact the capillaries and are structurally and functionally polarised. This indicates it is an important aspect of beta cell biology and needs to be incorporated into our models of human beta cell behaviour. Further work is needed to understand the role of beta cell polarisation and any relevance to improving cell-based treatments for diabetes.

## Supplementary information

ESM (PDF 1126 kb)

## Data Availability

All data generated or analysed during this study are included in the article. No additional resources were generated or analysed during the current study.

## Abbreviations

Dlg: Discs large
FAK: Focal adhesion kinase
nPOD: Network for Pancreatic Organ donors with Diabetes
Par3: Partitioning defective 3 homologue
RIM2: Rab3-interacting protein
