# Supplementary material for: Structural and functional polarisation of human pancreatic beta cells in islets from organ donors with and without type 2 diabetes
Source: Diabetologia. 2021 Jan 5;64(3):618–29. doi: 10.1007/s00125-020-05345-8 (PMC7864831; doi:10.1007/s00125-020-05345-8)
Supplement: Supplementary file 1 — (PDF 1126 kb) [file 125_2020_5345_MOESM1_ESM.pdf]

Electronic Supplementary Material (ESM)

Cottle et al. Structural and functional polarisation of human pancreatic beta cells in islets from organ donors with and without type 2 diabetes

ESM Tables

ESM Table 1: Characteristics of human pancreatic tissue donors

**Sample ID**	**Age**	**Sex**	**Source**	**Pancreatectomy or donor**	**Diabetes (Y/N), type and duration**	**Figure**
Sample 1	49	F	Royal North Shore Hospital	Partial Pancreatectomy	N	2
Sample 2	64	F	Royal North Shore Hospital	Partial Pancreatectomy	N	1, 2, 3, 4
Sample 3	73	M	Royal North Shore Hospital	Partial Pancreatectomy	N	1, 2, 3, 4
Sample 4	68	F	St Vincent's Institute	Cadaveric donor	N	1, 2, 3, 4
Sample 5	37	M	St Vincent's Institute	Cadaveric donor	N	1, 2
Sample 6	57	M	St Vincent's Institute	Cadaveric donor	Y, type 2, 8 years	6
6191	62.7	F	nPOD	Cadaveric donor	Y, type 2, 10 years	6
6283	57	F	nPOD	Cadaveric donor	Y, type 2, 17 years	6

ESM Table 2: Characteristics of human islet donors

Donor number	Diagnosed Diabetes	Age	Sex	BMI (kg/m^2)	Islet Source	Figure
1	No	37	F	32.4	St Vincent's Institute	5
2	No	64	F	23.5	St Vincent's Institute	5
3	No	41	M	37.32	Westmead Hospital	5
4	No	44	F	30.4	St Vincent's Institute	5

ESM Table 3: Details of diabetic samples

**Sample**	**Sample 6**	**6191**	**6283**
**Source**	St Vincent's Institute	nPOD	nPOD
**Diabetes duration**	8 years	10 years	17 years
**HbA1c**	7.2	6.0	9.6
**c peptide**	unknown	6.14	1.77
**BMI**	28.3	19.9	28.13
**Insulin dependent**	unknown	No	Yes
**Medication**	unknown	Metformin (1g/BID)	Liraglutide, Linagliptin, Insulin Detemir
**Histopathology**	unknown	Insulin+/Glucagon+ islets, multifocal, severe islet degeneration. Islet hyperplasia. Plugging of major ducts with high Ki67+ in epithelium. CD3+ infiltrates peri-ductal and exocrine regions, multifocal mild ductal mucinous dysplasia	Insulin+/Glucagon+ islets with severe amyloidosis. Moderate to severe acinar atrophy with multifocal chronic interstitial fibrosis and duct dilation (chronic pancreatitis). Severe fatty replacement within exocrine regions and interlobular regions. Abnormal PLNs PanBody. IPMN- branch duct (PanHead) with PanIN1-2 lesions other regions of intralobular ducts. PLN- histiocytosis

ESM Table 4: Antibodies used in this study

Antibody	Dilution	Source	Identifier
Anti-CD31	1:200	Abcam	Cat#Ab24590; RRID:AB_448167
Anti-Dlg	1:100	BD Biosciences	Cat#610874; RRID:AB_398191
Anti-ELKS (ERC1)	1:200	Abcam	Cat#Ab50312; RRID:AB_869944
Anti-glucagon	1:100	Merck	Cat#SAB4501137; RRID:AB_10761583
Anti-insulin	1:200	Dako Cytomation	Cat#A0564; RRID:AB_10013624
Anti-laminin (beta-1)	1:200	Pierce	Cat#MA5-14657; RRID:AB_10981503
Anti-liprin alpha 1 (PPFIA1)	1:200	Proteintech	Cat#14175-1-AP; RRID:AB_2171592
Anti-nidogen-1	1:200	R&D Systems	Cat#AF2570; RRID:AB_2282881
Anti-Par3	1:200	Merck	Cat#07-330; RRID:AB_11213581
Anti-piccolo	1:200	Synaptic Systems	Cat#142003; RRID:AB_2160182
Anti-RIM2	1:200	Synaptic Systems	Cat#140103; RRID:AB_887776
Anti-scribble	1:50	Santa Cruz	Cat#sc-11049; RRID:AB_2254275
Anti-syntaxin 1A	1:200	Synaptic Systems	Cat#110111; RRID:AB_887848
Anti-VWF	1:100	Sigma-Aldrich	Cat#F3520; RRID:AB_259543
Alexa Fluor 488 Donkey Anti-Guinea Pig	1:200	Jackson ImmunoResearch	Cat#706-545-148; RRID:AB_2340472
Alexa Fluor 546 Donkey Anti-Rabbit	1:200	ThermoFisher Scientific	Cat#A10040, RRID:AB_2534016
Alexa Fluor 594 Donkey Anti-Mouse	1:200	Jackson ImmunoResearch	Cat#A-21203, RRID:AB_2535789
Alexa Fluor 647 Donkey Anti-Rat	1:200	Jackson ImmunoResearch	Cat#712-605-153, RRID:AB_2340694

ESM Figures

ESM Fig 1

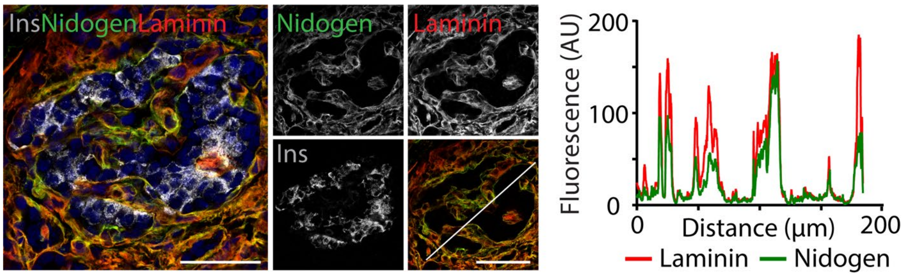

ESM Fig. 1 ECM proteins nidogen and laminin are co-localised in human pancreatic islets. Representative immunofluorescence images of a pancreatic islet demonstrating nidogen (green) is co-located with laminin (red). Linescan analysis demonstrates that the fluorescence of each protein target is enriched in corresponding areas within the islet. Scale bar: 50 µm. Ins, insulin

ESM Fig 2

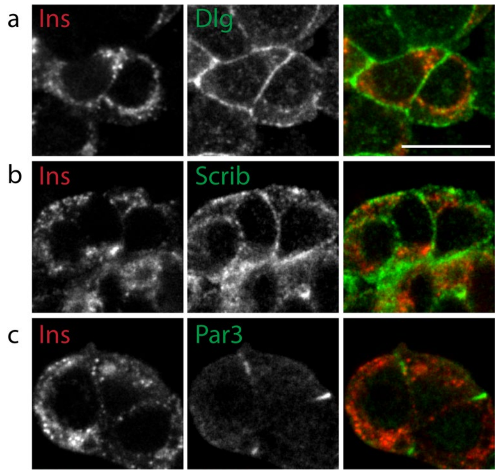

ESM Fig. 2 Human beta cells express polarity proteins Dlg, scribble and Par3. Representative immunofluorescence of dispersed beta cells cultured on laminin-511 stained with insulin (red), (**a**) Dlg (green), (**b**) scribble (green) and **(c**) Par3 (green). Scale bar: 10 μm. Ins, insulin; Scrib; scribble.

ESM Fig 3

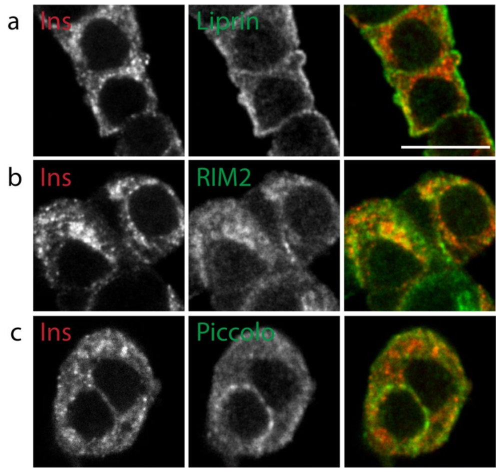

ESM Fig. 3 Human beta cells express presynaptic proteins liprin, RIM2 and piccolo**.** Representative immunofluorescence of dispersed beta cells cultured on laminin-511 stained with insulin (red), (**a**) liprin (green), (**b**) RIM2 (green) and (**c**) piccolo (green). Scale bar: 10 µm. Ins, insulin

ESM Fig 4

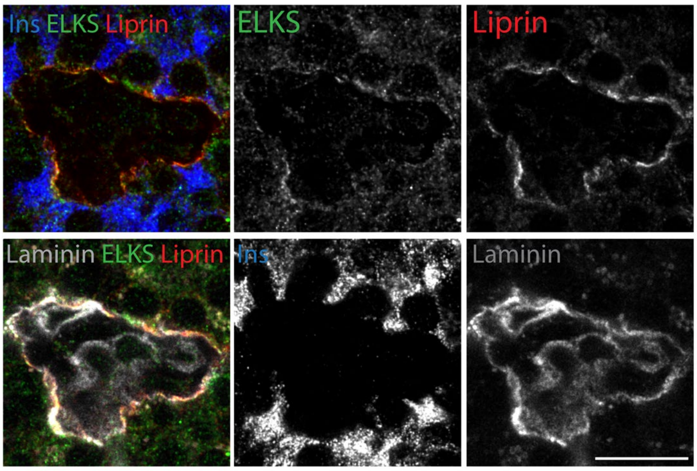

ESM Fig. 4 Both presynaptic proteins liprin and ELKS are enriched at the beta cell-vasculature interface. Representative immunofluorescence images showing beta cells (insulin- blue) surrounding an intra-islet blood vessel (laminin, grey). Presynaptic proteins liprin (red) and ELKS (green) are enriched along the vascular face of the beta cells. Scale bar: 20 µm. Ins, insulin

ESM Fig 5

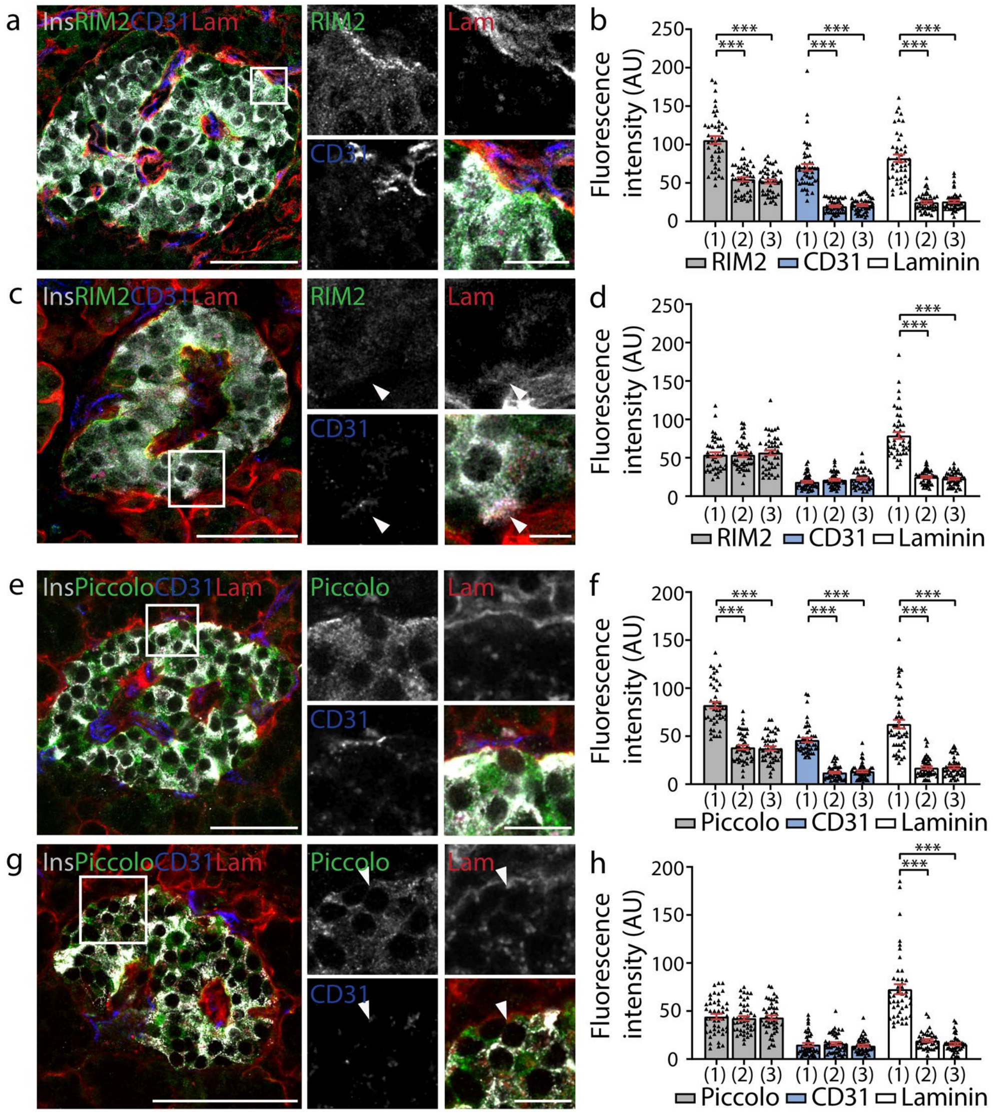

ESM Fig. 5 Presynaptic proteins RIM2 and piccolo are enriched along the vascular face of beta cells making contact with peri-islet blood vessels. **(a)** Representative images demonstrating RIM2 (green) is enriched along the peri-islet vascular face of beta cells (insulin- grey) on the outer edge of islets (laminin-red, CD31- blue). **(b)** Analysis of fluorescence intensity demonstrating enrichment of RIM2. RIM2 is not enriched along the islet capsule of beta cells on the outer edge of islets **(c)** Representative images, **(d)** Analysis of fluorescence intensity demonstrating a lack of RIM2 enrichment. Piccolo (green) is enriched along the vascular face of beta cells along the outer edge of islets. **(e)** Representative images **(f)** Analysis of fluorescence intensity demonstrating an enrichment of piccolo. Piccolo is not enriched along the islet capsule of beta cells on the outer edge of islets **(g)** Representative images, **(h)** Analysis of fluorescence intensity demonstrating a lack of piccolo enrichment. Data for both proteins (RIM2 and piccolo) are representative of 3 donors; n=45 cells, 15 cells from 3 islets per donor. Scale bars: 50µm on whole islet images and 10µm on zoomed-in images. Data were collected from both pancreatomized and cadaveric donor samples. *** $p<0.001$. Ins, insulin; Lam, laminin

Human Islet Checklist

Checklist for reporting human islet preparations used in research

Adapted from Hart NJ, Powers AC (2018) Progress, challenges, and suggestions for using human islets to understand islet biology and human diabetes. Diabetologia https://doi.org/10.1007/s00125-018-4772-2

Islet preparation	1	2	3	4	5	6	7	8[a]
MANDATORY INFORMATION								
Unique identifier	H278	SVI-024-18	SVI-027-18	SVI-004-20				
Donor age (years)	41	64	37	44				
Donor sex (M/F)	M	F	F	F				
Donor BMI (kg/m^2)	37.32	23.5	32.4	30.4				
Donor HbA_{1c} or other measure of blood glucose control	BGL 6.8	BGL 6.4-7.9 in ICU	BGL 6.5-11.7 in ICU	HbA1c 5.3 34mmol/mol				
Origin/source of islets[b]	Westmead Hospital	St Vincent's Institute	St Vincent's Institute	St Vincent's Institute				
Islet isolation centre	Westmead Hospital	St Vincent's Institute	St Vincent's Institute	St Vincent's Institute				

Donor history of diabetes? Please select yes/no from drop down list	No	No	No	No				
If Yes, complete the next two lines if this information is available								
Diabetes duration (years)								
Glucose-lowering therapy at time of death[c]								
RECOMMENDED INFORMATION								
Donor cause of death	SAH	Intracerebral Haemorrhage	Hypoxic brain injury	Traumatic brain injury				
Warm ischaemia time (h)	Unknown							
Cold ischaemia time (h)	366min	04:58	2:20	2:58				
Estimated purity (%)	80	70	95	90				
Estimated viability (%)	95		70	80				
Total culture time (h)[d]	14.5h	12.1h	10.9h	36h				

Glucose-stimulated insulin secretion or other functional measurement[e]	2.02							
Handpicked to purity? Please select yes/no from drop down list	No	No	No	No				
Additional notes	NA	NA	NA	NA				
